# Early infant diagnosis of HIV infection: a mixed-method study of uptake and challenges at primary health centers in Lagos State, Nigeria

**DOI:** 10.1186/s12913-023-09824-7

**Published:** 2023-09-28

**Authors:** BO Okusanya, CI Nweke, DO Akeju, J Ehiri

**Affiliations:** 1 Department of Obstetrics and Gynaecology, Faculty of Clinical Sciences, College of Medicine, University of Lagos, Idi-Araba, Lagos, Nigeria; 2 Department of Nursing Science, Faculty of Clinical Sciences, College of Medicine, University of Lagos, Idi-Araba, Lagos, Nigeria; 3 Department of Sociology, Faculty of Social Sciences, University of Lagos, Akoka, Lagos, Nigeria; 4 Department of Health Promotion Sciences, Mel and Enid Zuckerman College of Public, Health University of Arizona, Tucson, AZ United States of America

**Keywords:** Early infant diagnosis, HIV infection, Vertical HIV infection, perinatal HIV, MTCT of HIV

## Abstract

**Introduction:**

Nigeria has a low uptake of early infant diagnosis (EID) of HIV despite its high pediatric HIV infection rate. Efforts to increase the EID of HIV have been limited by many factors. This research assessed EID uptake and challenges service providers experienced in providing routine care for HIV-exposed infants.

**Methods:**

This is a mixed-method study at primary health centers (PHCs) in Lagos state, Nigeria. The quantitative component of the research was a review of the PMTCT Infant Follow-up Register at a purposive sample of 22 PHCs of Lagos State. The number of HIV-exposed infants (HEIs) returned for a dried blood sample (DBS) collection, date of collection, and the infant’s EID results for one year preceding the study were captured on Research Electronic Data Capture (RedCap). In-depth interviews were conducted with service providers purposively selected per participating PHC. Electronic transcripts were analyzed using MAXQDA 2020 (VERBI Software, 2019).

**Results:**

Twenty-two Lagos State primary health centers participated in the research. Fifteen PHCs (68.2%) had PMTCT HIV counseling and Infant follow-up registers. Documentation of DBS sample collection was observed in 12 (54.6%) PHCs. Both DBS sample collection and EID results documentation were observed in only nine (40.9%) PHCs. In-depth interviews revealed both maternal and health systems’ challenges to EID. The denial of HIV status was the only maternal factor reported as a barrier against the use of EID services. Health systems challenges include unavailability of EID services, uncertainty regarding whether EID is performed in a facility, referral to secondary health facilities for EID services (leading to losses to follow-up), and delay in getting results of EID. Task-shifting of DBS collection by nurses was suggested as means to increase access to EID services.

**Conclusions:**

There is a need to expand EID services and address women’s denial of HIV infection. Counseling women and linkage to available services are emphasized. Re-training of health workers on DBS collection and proper documentation of EID services were noted as key to improving the implementation of early infant diagnosis of HIV in the state.

**Supplementary Information:**

The online version contains supplementary material available at 10.1186/s12913-023-09824-7.

## Introduction

Although Nigeria’s prevalence of HIV infection is declining compared to figures a decade earlier, it has a persistently high rate of mother-to-child transmission (MTCT) of HIV infection of about 27% [1]. The magnitude of new pediatric HIV infection in Nigeria has made it one of the priority countries for the elimination of MTCT of HIV infection [1]. To achieve this, resources have been made available to support the national prevention of MTCT (PMTCT) of HIV infection [1].

Early infant diagnosis (EID) of HIV is recommended for all infants of women living with HIV infection, who are also referred to as HIV-exposed infants (HEIs). The World Health Organization (WHO) refers to EID as “virological testing using HIV DNA PCR and/or HIV RNA (PCR or other methods)” [2]. EID is mainly performed using polymerase chain reaction (PCR) techniques at 4–6 weeks of age and requires the mother to return her child to the health facility for the test [3]. Unfortunately, many HEIs do not perform the test for several reasons. Reported associations for poor EID service uptake include maternal factors such as maternal loss to follow-up, maternal non-adherence to combined antiretroviral therapy (cART), infant not on prophylaxis, maternal denial of HIV positive status, intimate partner violence, and maternal young age [4–8]. Also, health system factors reported as associated with low EID service uptake include inadequate staff with EID sample collection skills, unavailability of vehicles to convey samples to reference laboratories, and broken-down PCR machines [4]. Distance between the cART center and PCR facility, inadequate training, knowledge, and understanding of EID by service providers are other factors associated with low EID service uptake [7, 8]. For instance, a study on EID uptake in Myanmar reported that only 47% of HEIs had timely uptake of EID service at less than 8 weeks of age [8]. In Nigeria, HEIs were lost to follow-up in the PMTCT cascade of interventions before EID testing. Of infants who had EID, 77% had received results 28 months after sample collection [9]. Similarly, only 47% of EID results were received within 3 months of birth in Ethiopia [10].

EID performed after 8 weeks of age has been reported to be more likely to yield a positive result because infected infants have not had the appropriate treatment [11]. This is supported by an MTCT rate of 1.2% when the majority of HEIs had their tests within 8 weeks compared with an MTCT rate of 2.3% and 9% when the median age of testing was 17 weeks of age [11–13]. Prompt uptake of EID service is influenced by maternal disclosure of HIV status, cART adherence after birth [6], and mentor-mother support [14]. Other maternal factors associated with prompt EID service uptake include maternal formal education attainment and independent income source [13]. Health system facilitators of prompt EID service uptake include adequate knowledge of providers on EID, availability of DBS cards, and adoption of task-shifting strategies [4].

The prevailing low EID uptake of 12% in Nigeria and the relatively high HIV prevalence of Lagos state, compared with other states of Nigeria, informed the study [1, 15]. More so, a recent systematic review of interventions to increase uptake of early infant diagnosis of HIV infection concluded that there was no evidence that any existing interventions increased EID uptake at 4–8 weeks, partly because of the small sample sizes of included studies and wide confidence intervals of the effect size of the interventions [16]. The objectives of this study were to assess the uptake of EID and to identify challenges experienced by service providers in providing routine care to pregnant women living with HIV infection.

## Materials and methods

**Fig. 1:**
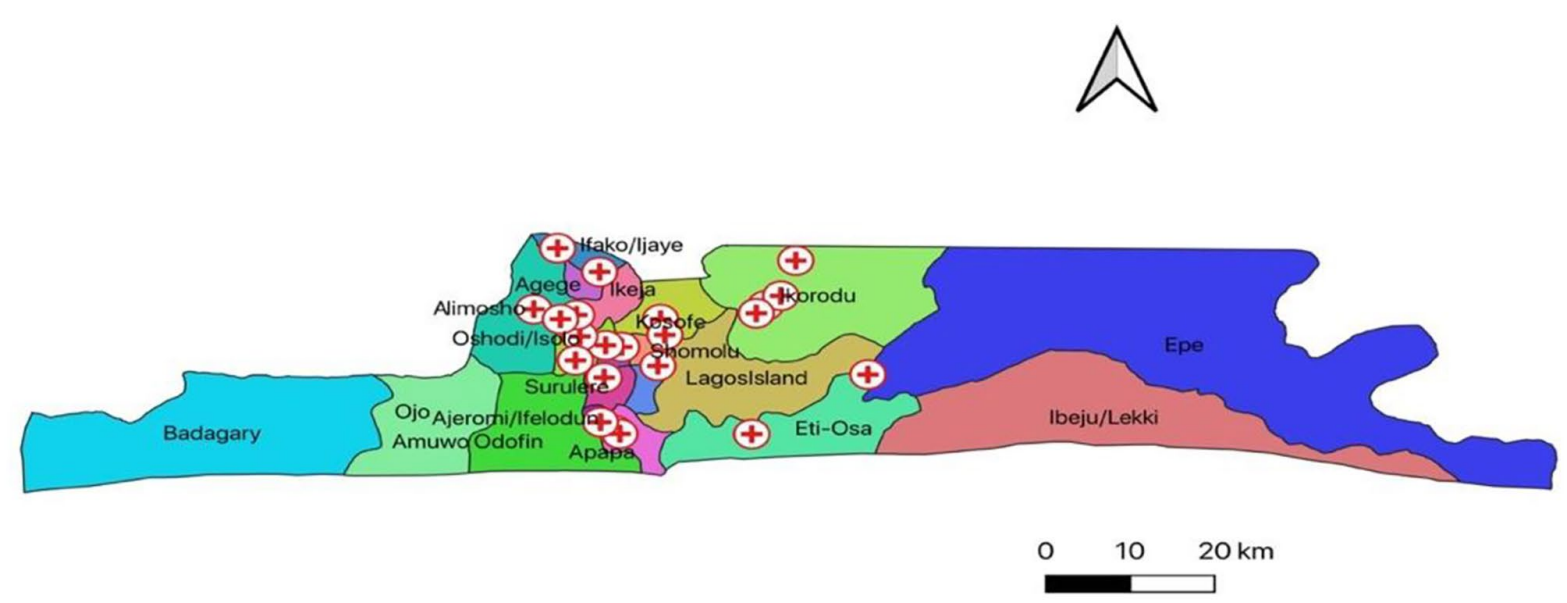
Map of location of participating primary health centers

Twenty-two (22) PHC centers were selected across four administrative districts of Lagos State, based on the high HIV prevalence of the local government areas (LGAs) in which the health facilities are located. The Lagos State Primary Health Care Board (LSPHCB) approved the research and provided a comprehensive list of PHC centers that offer 24-hour services in the state. Of the 78 PHC centers offering 24-hour services, 22 were selected because the LGAs had a record of 9 -121 pregnant HIV-infected women who had PMTCT interventions in 2020. See Fig. 1 for the map of the location of the PHC centers. By selecting facilities within communities with high HIV prevalence, it is assumed that service providers in them should have knowledge of the treatment guidelines and be using the same to care for HIV-infected women and their infants. Regular care of women with HIV infection has been reported to improve knowledge of treatment guidelines [17]. This also made participants more eligible to participate as they are more likely to encounter challenges when caring for HIV-infected women and their infants. Also, more nurses/midwives than physicians were approached to participate in the study as they are more in the employment of the LSPHCB.

### Data collection

Data were collected from July 28 to September 10, 2021. The quantitative component of the research was a document review of the PMTCT Infant Follow-up Register, one of the registers for PMTCT interventions. The register documents the uptake of EID services, including the date and age of DBS sample collection and the EID results. The number of HEIs returned for DBS sample collection, the date of the collection as well as the infant’s EID results were captured on Research Electronic Data Capture (RedCap) [18, 19] Uptake of EID services was assessed with the documentation of DBS sample collection and EID results for one year preceding the study team’s facility visit.

In-depth interviews were conducted with one service provider purposively selected per participating PHC to assess systemic issues related to EID service provision. Participants were nurses, midwives, and doctors who were trained to provide PMTCT services at participating PHC centers. These health workers were trained to offer prenatal care, screen pregnant women for HIV infection, and supervise childbirth, including newborn care. At each PHC center, the researchers approached PMTCT providers after the facility’s officer-in-charge (OIC) gave approval and permitted entry into the facility. In-depth interviews, which lasted 20 to 35 min each with selected service providers at participating PHCs, were conducted using an interview guide (Appendix 1). The interview guide encompasses PMTCT strategies like counseling and testing for HIV infection, provision of cART, viral load estimation, nevirapine prophylaxis, and HEIs feeding options. It was developed using specific tasks to be accomplished when providing the full spectrum of PMTCT services and was pilot tested in two non-participant PHCs. Also, participants were asked about the availability of EID services in their facility and if they experienced any challenges while providing any of the services. Participants in the qualitative research provided verbal consent.

### Data analysis

Interviews were recorded on digital voice recorders, and each recording was transcribed verbatim. Electronic transcripts were thereafter transferred to MAXQDA 2020 (VERBI Software, 2019) for data analysis [20]. Codes were derived deductively and independently by two researchers, a physician with in-depth medical terminology knowledge (coder 1) and a sociologist skilled in thematic content analysis (coder 2). By coding independently, the limitations imposed on the coding process by each person’s expertise were moderated by the strength of the other. After coding was completed, inter-coder reliability was assessed through a discussion between the two coders. All codes assessing early infant diagnosis were listed independently, with meaning well written out against each code. At the end of the exercise, four codes emerged for each independent coder: leading to 8 codes in all (Appendix 2). Of the 8 codes listed, 4 (50%) aligned with each other in terms of focus on early infant diagnosis and meaning; 4 (50%) varied significantly. This meant that only one person coding the transcript would have lost 50% of the codes. Thus, the approach enriched the data analysis process. Ultimately, themes and sub-themes were derived from the codes to report the qualitative research findings. Results were collectively reviewed by members of the research team, and findings were refined by linking themes and drawing further insight from the data.

### Ethical approval and consent to participate

Ethical approvals for the study were obtained from the Human Health Research Ethics Committee of the Lagos University Teaching Hospital (ADM/DCST/HREC/ APP/4031) and the Institutional Review Board of the University of Arizona, USA (Protocol Number: 2,101,422,001). The Lagos State Primary Health Board (LSPHB) approved the research and provided a comprehensive list of PHC centers that offer 24-hour services in the state.

Written informed consent was provided by all participants. Participation in the study was voluntary, and no incentives were provided. Respondents had the right to decline participation or to withdraw from the study at any stage if they wished. All methods were carried out in accordance with relevant guidelines and regulations.

## Results

**Fig. 2:**
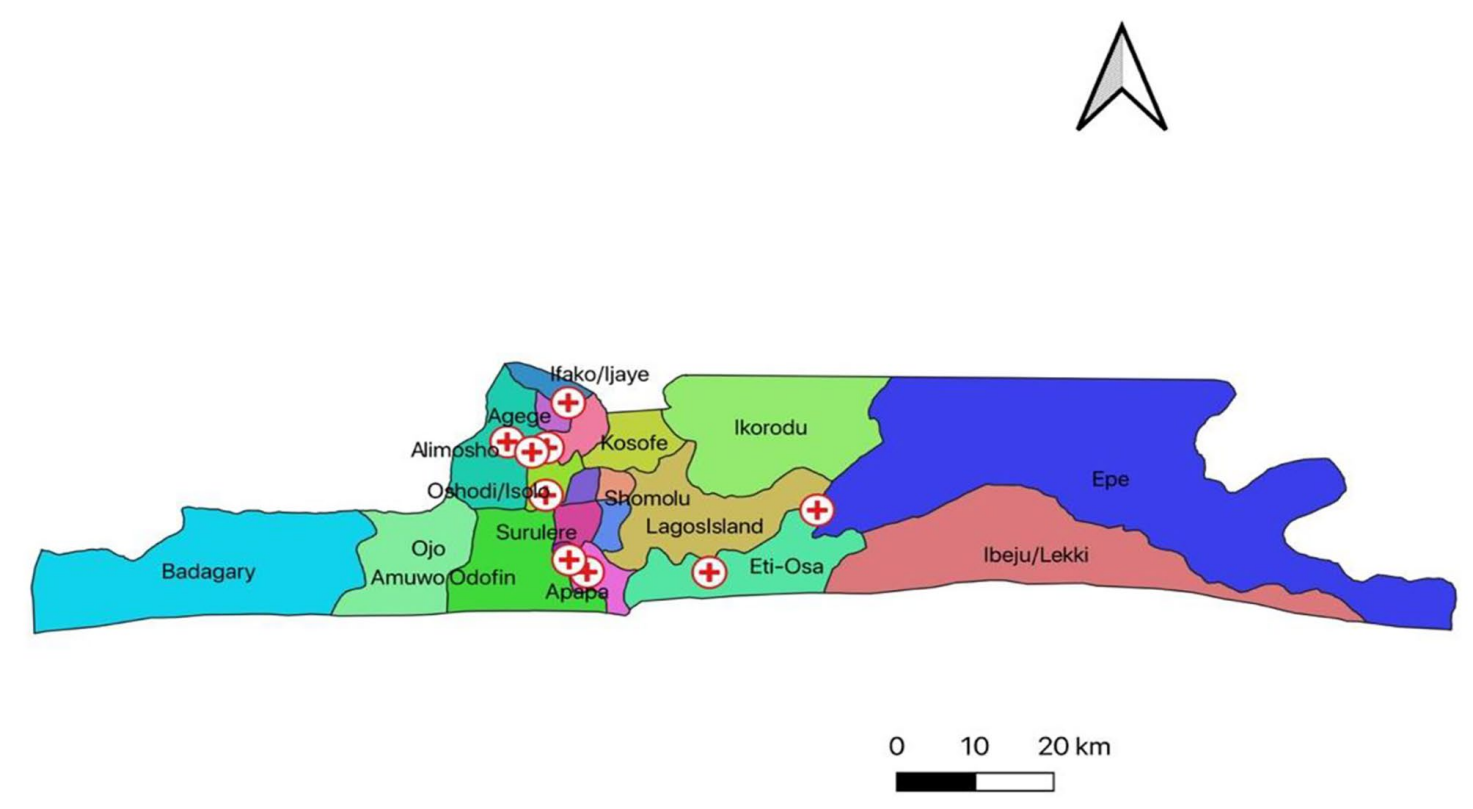
Location of PHC facilities with EID result documentation

Twenty-two Lagos State primary healthcare centers participated in the research. Seven (31.8%) PHCs had no PMTCT Register, though HIV screening was performed and documented with other investigations in the laboratory. Fifteen (68.2%) PHCs had both PMTCT HIV counseling and Infant follow-up registers. Thirteen facilities (59%) documented HIV screening in the context of PMTCT. Documentation of DBS sample collection was observed in 12 (54.6%) PHCs, while both DBS sample collection and EID results documentation were observed in only 9 (40.9%) PHCs. Figure 2 shows the map of the location of PHCs with EID result documentation.

Twenty-two service providers comprising 18 nurses/midwives and four physicians, participated in the in-depth interviews. Amongst the nurses/midwives, 10 (56%) were OICs, managing the facilities. Participants were mainly females (82%). Themes from the interviews include:

### Uptake of early infant diagnosis of HIV

The uptake of EID of HIV was enhanced by maternal counseling by service providers and the timing of EID to coincide with other routine services.

Qualitative data on early infant diagnosis of HIV showed that some children were returned by their mothers for DBS sample collection at 6 weeks of age. Data also showed that mothers were concerned about their children being HIV-infected, which made them return their children for the post-natal appointment for EID. Six participants (4 OICs and 2 nurses/midwives) reported uptake of EID services at their interviews.

“The women do come, and we don’t have a problem with them. This is because the women do not want their children to be HIV-infected. We inform the women of when to return their children for the test at 6 weeks. (CNO, PHC 4)

Compliance with uptake for EID service was influenced by consistent counseling and education of mothers by health workers. Also, the need to return to the health facility to access medications for their children and childhood immunization was a significant determining factor in compliance with the uptake of EID services. So, health workers leveraged routine services to deliver EID services to children.

“The babies do their DBS. I think that is basically what they do for them, as they are also commenced on Septrin® (Cotrimoxazole) at 6 weeks” (Nursing Officer, PHC 5)

*“After taking immunization at 6 weeks, the women bring the baby to run the test”* (Nurse/Midwife, PHC 10)

*“At 6 weeks, the women usually come with their babies for DBS in the lab. Then they are given the (Penta 1, Pc 1, Ib 1) immunization, which is compulsory, and we weigh the baby”* (CNO, PHC 4)

### Challenges of early infant diagnosis of HIV

Maternal and health systems’ challenges to early infant diagnosis of HIV infection were reported by participants.

#### Maternal challenges of early infant diagnosis of HIV

The denial of HIV infection by the mother was the only maternal factor reported to militate against the utilization of EID services. This was reported by a physician, who said:

“At times, due to loss to follow up or maternal denial of HIV infection because the woman believes that she is not HIV-infected, the baby is not brought back for the test” (Medical Officer, PHC 6)

#### Health systems challenges of early infant diagnosis of HIV

Participants reported a few health systems challenges of EID services within Lagos state’s primary health care systems. Some challenges mentioned include the *unavailability of EID services*, the uncertainty of EID service provision in their facilities, delayed EID result availability, and a potential for the infants to be lost to follow-up.

#### Unavailable EID services

Some PHCs do not provide EID services, including BDS collection. This was reported by five (3 OICs and 2 nurse/midwife) participants.

We don’t do DBS. It is not available; we only make the diagnosis antenatally and follow them up with their RVD and the baby.

(OIC, PHC 16)

No, we don’t collect DBS here

(Midwife, PHC 11)

#### Uncertainty of EID service provision and DBS collection timing

Two participants were unsure if their PHC provides EID services and the timing for DBS collection. A midwife reported:

*“No, this is a primary health care center I don’t think we do the test”.*

(Midwife, PHC 6)

An officer-in-charge of a PHC said: “6 weeks or, I am not very sure, I think it’s between 6 weeks and 6month”

(OIC, PHC 6)

#### Delay in receiving EID results

Two participants reported prolonged time from DBS sample collection to receipt of EID results as a challenge. When asked if the results of EID are returned early, a respondent reported:

No, because most times they send the sample to the general hospital, and the results come back late

(OIC, PHC 3)

When the dried blood samples are not properly taken as there should be three good samples of the five sent to the Laboratory, then there wouldn’t be an early result

(Medical Officer, PHC 6)

#### Referral to secondary health facilities for EID services

Some participants reported that the PHC refers HEIs to nearby secondary health facilities for EID services. These were facilities where either DBS collection services were not available, or the capacity to provide such services was weak. As such, referral became the means of closing this gap. Nevertheless, not all facilities utilize referral services. Three (2 OICs and 1 physician) participants reported they refer infants for EID services.

“No, we don’t lose the children to follow up. We follow them up as they go to the general hospital to do the test. As they will still bring the baby for vaccination, we use that opportunity to follow up on the baby”.

(OIC, PHC 16)

### Potential for infant loss to follow-up

An accompanying challenge associated with referral is loss to follow-up. While follow-up appeared effective in some facilities, data showed that some health centers do not have an effective follow-up system that ensures that women referred got to the point of referral. The essence of this deduction was captured in a statement by one of the senior health personnel (OIC, PHC 15) when she said: “*When they bring the child for immunization, we refer them to the general hospital for DBS. Once referred, we don’t have any other information on them”* This infers a potential loss to follow-up and of an opportunity for treatment.

In the face of the challenges, a participant made a case for task-shifting of DBS collection and stated:

I wouldn’t know. The laboratory staff has to be trained to collect DBS. However, the nurse may be trained to collect DBS

(IDI_CNO_PHC 16)

## Discussion

The objectives of this study were to assess the uptake of EID services and constraints encountered by service providers in the routine provision of EID services. Findings from the document review indicated that not all participating PHCs offered DBS sample collection, and only 41% of facilities had documentation of both BDS sample collection and EID results of HEIs. These findings were corroborated by participants’ responses on the uptake of EID services in the in-depth interviews, which also provided some insights to better understand the uptake and challenges of EID of HIV at the primary health centers in Lagos State.

Constraints of EID services experienced by service providers included maternal denial of HIV infection, unavailability of EID services, provider’s poor knowledge of the timing and availability of EID services, referral to secondary health facilities, and delayed EID results.

This research documents HIV-EID deserts in Lagos State, Nigeria, with a rather low proportion of PHCs that provide DBS sample collection and EID results documented. This finding reflects the challenges of EID of HIV in many countries of sub-Saharan Africa [7, 9, 10, 12]. The gradual reduction in the number of facilities that render EID services, compared with those which provide HIV counseling and testing services in pregnancy, implies that some women might need to travel some distance to access EID services for their HEIs. The long distance between facilities that provide PMTCT services and EID services has been reported as a challenge to EID service uptake and early return of results [8, 21]. There is a need for all PHCs that provide intrapartum care for women living with HIV to provide DBS sample collection services at a minimum.

Maternal denial of HIV infection is a challenge in PMTCT strategies that is not limited to EID service uptake. Denial of HIV infection has been reported to affect every PMTCT cascade of care intervention negatively. For instance, maternal denial of HIV status has been reported to reduce maternal initiation of cART in pregnancy and is associated with poor cART compliance or an outright stoppage of treatment [10, 22]. Maternal denial of HIV infection has significant implications on her infant’s health as her infant might not be returned for EID services, making it impossible to diagnose an infected child early enough to commence treatment. This has a risk of higher infant mortality for HEIs. Other studies have reported maternal denial of HIV status as a challenge to EID services uptake [4, 8]. To reduce the occurrence of denial of HIV status, service providers should counsel pregnant women newly diagnosed as HIV-infected on why they should come to terms with the diagnosis using data of successful PMTCT outcomes, like the proportion of HEIs who have negative EID results and remain healthy so that they are encouraged to take cART and comply with other instructions of the health workers.

While poor knowledge of PMTCT interventions by service providers has been reported as a constraint to PMTCT, including EID services [4], this study brings to the fore the uncertainty of the timing and availability of EID services in the same facility that the providers rendered services. Delayed EID uptake has been reported to be more likely to be associated with infant HIV infection. Hence the need for training/refresher training for service providers on the timing of critical and specific PMTCT interventions. Also, service providers should be knowledgeable about available services, including EID services, offered by their facilities.

Delayed return of EID results to the facility and maternal notification of results is a major challenge of PMTCT in Nigeria and many SSA countries. Delayed results may be due to several reasons, including poor sample collection, batching of samples to be sent to the reference laboratory, malfunctioning PCR machines, and workload of staff operating the machine in the reference laboratory [4]. Participants reported poor DBS sample collection technique and the use of reference laboratories of secondary health facilities as the reasons for delayed EID results availability.

An important consideration of scaling up PMTCT services to PHCs in Nigeria was the proximity to a secondary or tertiary health facility with personnel who might provide support to PHC service providers to render quality care for women living with HIV infection in pregnancy [23]. Referral of HEIs to secondary health facilities for EID services was well-thought support for the PHC systems in Lagos state. However, service providers were unable to verify if the women and their HEIs made use of the referral. Also, they reported that referral likely caused maternal and infant loss to follow-up in the postpartum period. When EID services were performed at secondary health facilities, there was also delayed result release to the PHCs, which ultimately caused a delay in the commencement of treatment for HIV-infected infants.

The strength of this research is the use of an objective measure of documentation in the PMTCT infant follow-up register to determine the uptake of EID services. The study was conducted at 24-hour PHCs that rendered antenatal and delivery services across four administrative districts of Lagos state, using LGAs with high HIV prevalence. Also, there was a corroboration of the findings of the document review by participants’ responses to the in-depth interviews. The use of two researchers who independently coded the themes from the interview transcripts ensured a rich qualitative data analysis. Despite these, the study is limited to using an interview guide to facilitate the interviews, which might have restricted the scope of participants’ responses. The impact of this was minimized by the use of prompts and follow-up questions to participants’ responses. Also, only 22 health facilities participated, and findings might not reflect the state of EID services across Lagos state. The study, however, provided insight into a national debacle.

### Implications for practice and policy

More efforts and newer strategies will be required to improve the rather low 12% EID uptake in Nigeria [1]. Strategies should be tailored to challenges identified with EID service uptake. Maternal denial and the need to provide comprehensive post-test counseling have been discussed earlier.

To make EID services more accessible, all 24-hour PHCs rendering maternity services for HIV-infected women should be supported to collect DBS. This will reduce the need for referral to another facility and reduce the potential for loss to follow-up of HEIs. Due to the technique and skills of DBS collection, sample preservation, and transport to the reference laboratory, there is a need to retrain and train more service providers to avoid delays in getting EID results or the inability to process samples. Training will also make service providers know the timing of DBS collection.

Task-sharing of DBS collection by nurses/midwives with laboratory technicians should be explored since more nurses/midwives work in the PHCs. In this light, consideration should be given to expanding the roles of nurses who provide childhood immunization services to render DBS collection. There should be monitoring and technical support for all service providers involved in DBS collection, and relevant PMTCT program registers, including the Infant Follow-up Register, should be supplied to every facility that provides EID services for documentation. Registers that have been filled out should be safely archived for future reference.

## Conclusion

The uptake and constraints of EID services in selected Lagos state PHCs reported in this study highlight the situation in participating PHCs. With maternal HIV denial as the only maternal constraint to EID services, efforts to persuade women newly diagnosed with HIV infection to come to terms with the infection should be emphasized during post-testing counseling and the period following maternal HIV diagnosis. Measures should be put in place to address the facility-based constraints to EID services, such as but not limited to re-training of health workers on the timing of EID services as an integral component of PMTCT interventions is advocated.

### Electronic supplementary material

Below is the link to the electronic supplementary material.

Supplementary Material 1

Supplementary Material 2

## Data Availability

The datasets generated and analyzed during the current study are not publicly available because they are being used for secondary analysis and more publications. However, they are available upon a reasonable request to the corresponding author.
