# Supplementary material for: Early infant diagnosis of HIV infection: a mixed-method study of uptake and challenges at primary health centers in Lagos State, Nigeria
Source: BMC Health Serv Res. 2023 Sep 28;23:1038. doi: 10.1186/s12913-023-09824-7 (PMC10536780; doi:10.1186/s12913-023-09824-7)
Supplement: Supplementary file 1 — Supplementary Material 1 [file 12913_2023_9824_MOESM1_ESM.pdf]

Appendix 1: Interview Guide

Objective: To assess barriers to guideline implementation by health workers in routine clinical practice using in-depth interviews.

Courtesy: Good morning/ afternoon,

I am here to ask you about your experience when taking care of HIV-positive pregnant women. My questions will cover antenatal, delivery and postpartum care. I will be recording our discussion with a voice recorder.

Do you permit me to talk to you and record the discussion?

If yes,

Can you please introduce yourself, the work you do and where you work?

Questions:

1. Can you share your experience on what you do when you attend to a pregnant HIV-positive woman?
   Prompts:
   - Do you use any job aid/ charts/ pictures to guide what you do?
   - Do you have seminars/ in-house trainings on what to do?
   - How regular are the trainings if you have seminars/ trainings?

2. As with any role, challenges may occur when we perform our roles. Can you share your experience about the challenges you encounter when you attend to a pregnant HIV-positive woman?

   Prompts: (PLEASE INTERVIEWER SHOULD ENSURE TO PROBE FOR CAUSES FOR EACH CHALLENGE MENTIONED)
   - What is/are the cause(s) of such challenge? (Individual and institutional factors)
   -
3. When you have a challenge or unsure of what to do, how do you solve the problem?

   Prompts: (INTERVIEWER SHOULD ENSURE TO PROBE FOR EACH SOLUTION MENTIONED)
   - Do you ask your colleague or supervisors/ seniors?
   - Do you refer to job aid/ instructions/ guidebook/ treatment guidelines?
   -

4. What can you say is the difficulty you face in the facility when you care for an HIV-positive woman in late pregnancy?
   Prompts:
   - Do you repeat HIV screening?
   - What are the difficulties involved when you repeat HIV screening?
   - Do you assess viral load or refer to a place where viral load estimation may be performed?
   - What are the difficulties involved when you assess viral load or refer to a place where viral load estimation?

5. Do you have a copy of the HIV PMTCT Guideline in this facility?
   Prompts:
   - If yes, (Ask to see a copy)
   - have you read it before? When was the last time you read it?
   - Is it in the form of a booklet/ charts/ pictures?

6. Do you screen all pregnant women for HIV infection in this facility?
   Prompts:
   - Who does the screening?
   - Any pre-test counselling? How is it done?
   - Any post-test counselling? How is it done?
   - Do you refer elsewhere for HIV testing?
   - How soon are results made known to pregnant women?
   - If someone tests positive to HIV infection, what do you do?
   - On the average how soon does a woman with HIV positive result start HIV medications
   - What are the challenges encountered by health workers when you screen pregnant women for HIV?

7. Do you give HIV medications to pregnant women in this facility?
   - If no, how do your patients get their HIV medications
   - Are there challenges with HIV medications among pregnant women with HIV

8. Let’s consider a typical scenario when an HIV-positive woman is giving birth. Can you share your experience about how you care for her?

   Prompts:
   - Do you delay ARM (Artificial rupture of membrane)
   - Do you give episiotomy (Cut on the vaginal outlet) always?
   - Do you use the partogram to monitor them during childbirth?

9. What is your experience in giving infant prophylaxis (Nevirapine syrup) to babies of HIV-positive women?
   Prompts:
   - Experience about storing Nevirapine syrup in the facility?

- Experience about the correct dose to give.
- Challenges about giving infant prophylaxis (Nevirapine syrup) to babies of HIV-positive women

10. Can you share your experience about how babies born to HIV-positive mothers in your facility are fed?

11. After discharge of a HIV-positive mother and her baby, do you ask her to bring back her child?

- When do you ask her to bring back her child?
- Are there some challenges encountered by HIV-positive mothers when they are asked to bring their babies back to the health centre?

12. Do you collect blood (dry blood sample, DBS) for early infant diagnosis of HIV in this facility?

Prompts:

- When do you tell mothers to bring back their child for DBS?
- What are the challenges involved or encountered in doing this?

Lastly, are there other problems you face, that we have not discussed, that you want to mention now?

Closing remarks:

Thank you for your time. The interview has ended
