# Supplementary material for: Early infant diagnosis of HIV infection: a mixed-method study of uptake and challenges at primary health centers in Lagos State, Nigeria
Source: BMC Health Serv Res. 2023 Sep 28;23:1038. doi: 10.1186/s12913-023-09824-7 (PMC10536780; doi:10.1186/s12913-023-09824-7)
Supplement: Supplementary file 2 — Supplementary Material 2 [file 12913_2023_9824_MOESM2_ESM.pdf]

Appendix 2: Codes generated deductively and their meanings

Coders	Serial No.	Codes	Meaning	Number of transcript quotes
Coder 1	1.	DBS Sample Collection	The process of collecting, sending and retrieval of result and any challenges associated with the process	32
	2.	Non-disclosure of HIV status	A situation where women refuse or show reluctance to disclose their status to their spouse. It also includes women forbidden health workers to disclose their status to their spouse	13
	3.	Denial of HIV result	Refusal to accept HIV infection status	8
	4.	Referral to secondary facility	When HIV positive mothers or their children are referred to another health facility to continue treatment for HIV in the context of early infant diagnosis	23
Coder 2				
	5.	Challenge_DBS	The challenges involved in the collection of dry blood sample as well as the process and system that support it	1
	6.	Referral	Linking women up with another health facility to access care because of lack of expertise/equipment or because the facility is not empowered to offer such services	3
	7.	Care for infant_DBS	All types of care rendered to infant children in the process of collecting their dry blood sample	15
	8.	Postnatal appointment	All appointments given to women with positive HIV status shortly after delivery, including appointment for vaccination and DBS	10
